# Soil phosphorus mediates trade-offs between constitutive and induced defences in young pine trees

**DOI:** 10.1007/s00425-025-04813-y

**Published:** 2025-08-28

**Authors:** Xoaquín Moreira, Luis Abdala-Roberts, Rafael Zas, Luis Sampedro

**Affiliations:** 1 Misión Biológica de Galicia (MBG-CSIC), Apdo. 28, 36080 Pontevedra, Galicia, Spain; 2 Departamento de Ecología Tropical, Campus de Ciencias Biológicas y Agropecuarias, Universidad Autónoma de Yucatán, Apartado Postal 4-116, Itzimná, 97000 Mérida, Yucatán México

**Keywords:** Jasmonic acid, Nutrient availability, Phenolic compounds, *Pinus pinaster*, Resin

## Abstract

**Main conclusion:**

Nutrient availability, namely soil phosphorus, modulates trade-offs between constitutive and induced defences in maritime pine, with high phosphorus weakening these trade-offs and shaping plant allocation to different defensive strategies.

**Abstract:**

Abiotic factors modulate trade-offs between plant functions, but their influence on trade-offs between constitutive and induced defences remains poorly understood. We tested for such trade-offs in maritime pine (*Pinus pinaster*) and examined whether soil phosphorus availability affected these defensive correlations. We conducted a greenhouse experiment with six-month-old pine seedlings from 33 half-sib families, exposing half of the plants from each family to either low or high soil phosphorus availability, one of the main limiting factors for pine development in the study region. Within each fertilization group, we applied jasmonic acid to induce defences in half of the plants per family. Defensive traits measured included resin production and phenolic compound levels. As predicted, we found significant negative correlations between constitutive and induced defences for both defensive traits under low phosphorus availability. However, these correlations were absent under high phosphorus conditions, indicating that overabundance of this nutrient weakened defensive allocation constraints. These findings highlight the role of nutrient availability in shaping plant defence allocation constraints, potentially shaping the correlated evolution of plant defensive strategies.

**Supplementary Information:**

The online version contains supplementary material available at 10.1007/s00425-025-04813-y.

## Introduction

Plants and insect herbivores have interacted for over 500 million years (Labandeira 2007; Kergoat et al. 2017), resulting in a remarkable diversity of defence mechanisms on both sides (Futuyma and Agrawal 2009). Plant defences are broadly classified as constitutive or induced: constitutive defences represent baseline levels that provide continuous protection, whereas induced defences are triggered by herbivore damage, allowing for more efficient and targeted investment (Núñez-Farfán et al. 2007). A key insight in plant defence research is that constitutive and induced defences often trade off and, therefore, do not evolve independently (Stamp 2003; Rasmann et al. 2015; Agrawal and Hastings 2019). These trade-offs arise from resource allocation constraints, leading to negative correlations between these defensive strategies (Kempel et al. 2011; Moreira et al. 2014).

Trade-offs between constitutive and induced defences are well documented both across and within species, especially in herbaceous plants (Bingham and Agrawal 2010; Kempel et al. 2011; Rasmann and Agrawal 2011; Rasmann et al. 2015; Agrawal and Hastings 2019; Kalske and Kessler 2023). However, these patterns may not directly apply to trees, which differ substantially in growth form and life-history traits that shape resource allocation and defence strategies. Trees grow slowly, are long-lived, and store reserves in woody tissues, necessitating durable constitutive defences over extended periods (Massad 2013). In contrast, fast-growing, short-lived herbaceous plants often rely more on rapid, inducible chemical defences (Massad 2013). Despite these fundamental differences, defensive trade-offs in trees remain poorly studied (but see Moreira et al. 2014; Villari et al. 2014), representing a key gap in our understanding of the diversity, mechanisms, and ecological and evolutionary foundations of plant defence strategies.

Abiotic factors influence trade-offs among key plant functions, including growth and defence (Züst and Agrawal 2017). The Resource Availability Hypothesis predicts that plants in resource-poor environments invest more in defences because tissue replacement is costly, often at the expense of growth (Coley et al. 1985; Endara and Coley 2011). While this prediction is supported for light and water availability (Züst and Agrawal 2017), to our knowledge, no previous study has directly tested how abiotic factors shape trade-offs between constitutive and induced defences (for growth-defence trade-offs see Sampedro et al. 2011). Clarifying whether nutrient availability constrains or modulates the simultaneous expression of constitutive and induced defences is essential for understanding how the abiotic context shapes defence allocation and the evolution of plant resistance.

*Pinus pinaster* Ait., commonly known as maritime pine, is a Mediterranean-native species renowned for its ecological and economic significance (Bucci et al. 2007). It is well adapted to coastal sandy soils, where it thrives under challenging abiotic conditions (Bucci et al. 2007) and plays a crucial role in stabilizing forest soils and other ecosystem functions as well as in sustainable forest management and restoration efforts (de la Mata et al. 2024). Among the abiotic stressors faced, soil nutrient limitation is particularly important, with phosphorus availability strongly constraining plant growth and influencing pine-associated ecosystem dynamics in Galician coastal forests (Martíns et al. 2009). Maritime pine produces chemical defences in its leaves and stems, especially phenolic compounds and resin (composed primarily of terpenoids), which deter and negatively affect the performance of phytophagous insects and pathogenic organisms (Mumm and Hilker 2006). Constitutive concentrations of resin and phenolics are particularly high in *P. pinaster* tissues, reaching levels of up to 200 mg per gram of dried tissue weight (Sampedro et al. 2011; Moreira et al. 2014). At the same time, these defences are highly inducible in response to herbivory and contribute to induced resistance against insect herbivores (Sampedro et al. 2011; Moreira et al. 2014). Previous work on *Pinus pinaster* has shown that when phosphorus is limited, plants significantly reduce their growth and increase their investment in chemical defences (Sampedro et al. 2011), but it is currently unknown whether phosphorus limitation affects the correlations between defensive strategies in these traits.

In this study, we examined potential trade-offs between constitutive defences and their inducibility in maritime pine (*Pinus pinaster*) seedlings, and assessed their dependence on soil resource availability—specifically phosphorus, a key limiting factor for pine development in the coastal habitats of Galicia, north-western Spain (Martíns et al. 2009). To achieve this, we conducted a greenhouse experiment with 33 half-sib pine families, for which we factorially manipulated defence induction by applying jasmonic acid (Moreira et al. 2014) and soil phosphorus availability via fertilization (high availability: 20 mg P L⁻^1^; low availability: 2 mg P L⁻^1^). We focused on two key defensive traits in pines: non-volatile resin in stems (the fraction that functions both as a physical barrier and a chemical deterrent to insect feeding) and total phenolics in needles (Sampedro et al. 2011; Moreira et al. 2014). A negative correlation between constitutive defences and their inducibility in each case suggests a defensive trade-off. We predicted that high phosphorus availability would weaken this correlation, presumably by alleviating allocation constraints between constitutive and induced defence expression. Findings from this work provide mechanistic insights into the abiotic control, particularly of soil nutrient availability, over plant allocation to constitutive and induced defences, ultimately enhancing our understanding of plant defence and, more broadly, life history evolution.

## Material and methods

### Plants and experimental design

Seeds from *Pinus pinaster* 33 half-sib families, obtained from the seed orchard of the Xunta de Galicia in Sergude (A Coruña, Galicia, NW Spain; 42.818261°N, 8.461284°W), were individually sown in 2-L containers filled with sterilized perlite. The seedlings were grown in a greenhouse under controlled conditions, with a minimum of 12 h of light per day and temperatures of 25 °C during the day and 18 °C at night. One month after sowing, half of the plants were subjected to one of two fertilization treatments through subirrigation every 2 days for 5 months: a complete nutrient solution and a phosphorus-deficient solution. The complete fertilizer treatment was formulated to meet the optimal growth requirements of *P. pinaster* seedlings and contained 100:20:70:7:9 mg L⁻^1^ of N, P, K, Ca, and Mg, respectively, along with several micronutrients, including Cu, Fe, Zn, Mo, B, and Mn (Sampedro et al. 2011). In comparison, the phosphorus-deficient treatment reduced phosphorus availability tenfold to 2 mg P L⁻^1^, while keeping the concentrations of other nutrients constant, thereby mimicking the low-P conditions typical of coastal sites in the region (Martíns et al. 2009). Both treatments were adjusted to a pH of 6.5. Fertilization began one month after sowing to allow seedlings to establish initial root systems under uniform conditions, minimizing early mortality and ensuring comparable starting points at the time of nutrient treatment application.

At 6 months of age, seedlings in the phosphorus-deficient treatment were significantly shorter than those in the complete fertilization treatment (*F*_1,32_ = 142.52, *P* < 0.001), averaging 22.6 ± 0.7 cm versus 47.8 ± 1.2 cm, respectively (see Suppl. Fig. S1). At this moment, we applied the induction treatment: half of the plants in each fertilization treatment received a foliar application of 22 mM jasmonic acid (JA) (Sigma-Aldrich, #39270-7) in deionized water containing 2.5% ethanol (v/v). The other half of the seedlings served as controls and were sprayed with the carrier solution only. The JA and control solutions were sprayed until runoff (approximately 2.6 ± 0.2 or 3.7 ± 0.3 mL for each phosphorus-deficient and complete fertilized plants, respectively). The JA concentration chosen and application procedure were adapted from protocols previously validated for *P. pinaster* (Moreira et al. 2009). To prevent cross-contamination, JA-treated and control plants were placed in separate rooms during treatment application, where they remained drying for 24 h before placing them back in their original rooms and randomized positions. The experiment followed a randomized split-split plot design (fertilization: plot level; induction: split-plot level; family: split-split plot level), where plants within the same fertilization treatment were spatially grouped due to irrigation constraints, and the remaining factors were randomized within each fertilization group. This design was replicated across four blocks, resulting in a total of 528 seedlings: 33 families × 2 fertilization treatments × 2 induction treatments × 4 blocks. Two weeks after JA application, all plants were fully harvested. Needles and stems were separated; needles were oven-dried at 40 °C for 48 h for phenolic analysis, and stem material was stored at −20 °C for resin analysis. All needles from each plant were pooled into a single sample for analysis, providing an estimate of whole-plant-level defence allocation.

### Chemical analyses

For the analysis of needle phenolics, we extracted phenolics from 300 mg of finely ground, dried pine needles using a 1:1 volumetric mixture of aqueous methanol in an ultrasonic bath for 15 min (Moreira et al. 2014). Following extraction, we centrifuged the mixture at 698.5 g and diluted the methanolic extract. The total phenolic content was then quantified colorimetrically using the Folin-Ciocalteu method, with a Biorad 650 microplate reader set to 740 nm and tannic acid as the standard, with results reported on a dry weight basis.

To estimate the concentration of non-volatile resin in the stem, we applied a gravimetric approach (Moreira et al. 2014). Approximately 5 g of fresh stem material was crushed and placed in pre-weighed tubes, each 20 cm in height. Resin compounds were extracted using hexane in an ultrasonic bath, and the extraction process was repeated. The solvent was evaporated, and the residue mass of non-volatile resin was measured to the nearest 0.0001 g, providing an accurate assessment of the resin concentration in relation to the stem’s dry weight.

### Statistical analyses

First, we obtained least square means for each family under control and induced conditions, separately within each level of the soil fertilization treatment. To do this, we ran linear mixed models for non-volatile resin in the stem and phenolic compounds in the needles, including phosphorus availability (P), induction treatment (T), and their interaction (P × T) as fixed factors. Family and its interactions with the main effects (P, T, and P × T) were also treated as fixed factors. We included the block effect (B) and its two-way interactions with the main effects (B × P and B × T) as random factors to ensure proper error terms for analysing the main effects. Although half-sib families are often treated as a random effect in quantitative genetic studies—given the interest in variance components and broader inference—here we treated family as a fixed factor. Our goal was solely to obtain family-level means within each fertilization × induction combination, accounting for any sample size imbalances, rather than estimating family means across other factor levels or quantifying variance components. All analyses were performed using PROC MIXED (SAS Institute, Cary, NC, USA) (Littell et al. 2006). The results from these models are presented in Sampedro et al. (2011).

Second, using the family means from the models described above, we conducted family-level Pearson correlations between constitutive levels and inducibility for each trait, separately within each fertilization treatment. Least square means for control plants of each family represented constitutive levels, while inducibility was determined by calculating the difference between the least square means of control and induced plants of each family. A negative correlation for a given trait would support the idea of a trade-off between constitutive and induced defences. However, this approach poses the challenge of including the same variable (i.e., the constitutive values) as both an independent variable and in the calculation of the response variable, potentially leading to spurious correlations (Morris et al. 2006). To circumvent this issue, we implemented a Monte Carlo simulation approach following Morris et al. (2006). We performed 10,000 simulations of constitutive and inducible resistance values using data distributions parameterized from the computed means and variances. This number of simulations was chosen to ensure stable estimates of the null distribution and associated *P*-values, as higher numbers of simulations further reduce sampling error but with diminishing returns in precision (Morris et al. 2006). For each simulation, we computed Pearson correlation coefficients and summarized the results as frequency histograms. From these, we derived Monte Carlo *P*-values and compared them to the observed Pearson *P*-values to discriminate true trade-offs from spurious correlations. This procedure was conducted in SAS by adapting the original MATLAB code of Morris et al. (2006) (see more details in Suppl. Fig. S2 and S3).

## Results

**Fig. 1:**
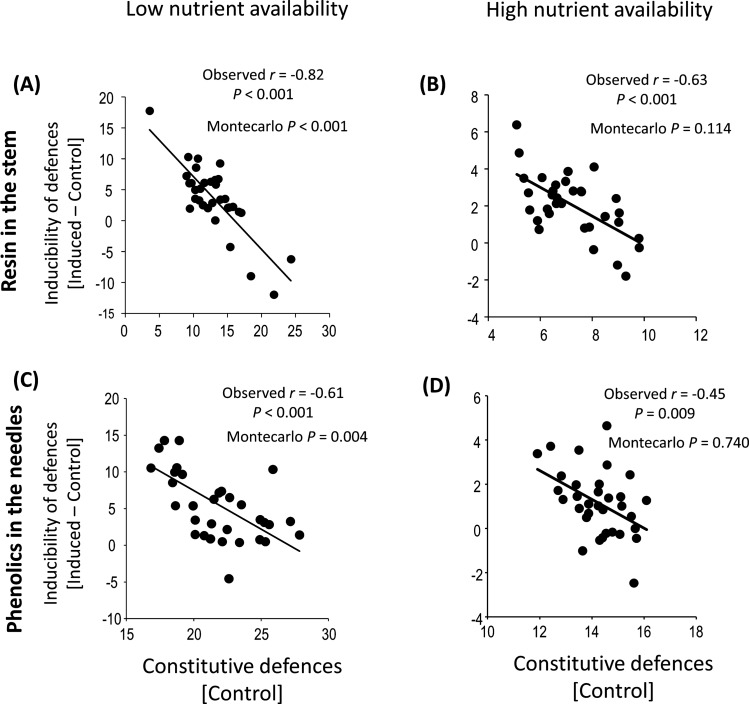
Plant family-level correlations between the constitutive concentrations (in mg g^−1^ DW) of non-volatile resin in the stem (**A**, **B**) or total phenolics in the needles (**C**, **D**) and their corresponding inducibility in *Pinus pinaster* seedlings, shown separately under low (*left side panels*) or high nutrient availability (*right side panels*). Inducibility was measured as the family mean values of jasmonic acid (JA)-induced plants minus the mean of control plants. Shown are Pearson correlation coefficients, whereby significant negative relationships denote a genetic trade-off between constitutive and induced defences. Monte Carlo *P*-values indicate the probability of obtaining a spurious correlation for the observed Pearson *r* and associated data error (see “Materials and methods”). Each point represents a half-sib *Pinus pinaster* family (*n* = 33), and all families were included in each correlation analysis

We found evidence that soil phosphorus availability affects family-level correlations between constitutive pine defensive traits and their inducibility. Specifically, under reduced phosphorus availability, significant negative, non-spurious correlations were observed between constitutive levels and inducibility for both non-volatile stem resin concentration (*r* = −0.82, *P* < 0.001, *n* = 40; Fig. 1A) and needle total phenolics (*r* = −0.61, *P* = 0.004, *n* = 40; Fig. 1C). In contrast, no significant associations were found under high phosphorus availability (stem resin: *r* = −0.63, *P* = 0.114, *n* = 40; Fig. 1B; needle phenolics: *r* = −0.45, *P* = 0.740, *n* = 40; Fig. 1D).

## Discussion

We found negative correlations between constitutive defences and their inducibility for two key defensive traits in *P. pinaster*: non-volatile resin in stems and total phenolics in needles. These results align with previous studies in herbaceous plants, such as *Asclepias* species (Bingham and Agrawal 2010; Agrawal and Hastings 2019), *Arabidopsis thaliana* (Rasmann et al. 2015), wild crucifers (Zhang et al. 2008), and various cultivated garden plants (Kempel et al. 2011). Similar patterns have also been observed in some tree species, including *Populus tremuloides* (Cole et al. 2021) and other *Pinus* species (Villari et al. 2014; Runyon et al. 2022). Although more research on trees is necessary to draw broader conclusions, current evidence suggests that negative correlations between constitutive and induced defences are common across diverse plant species. These patterns are likely driven primarily by resource allocation constraints, despite considerable variation in life-history traits such as growth form, longevity, and woodiness (Koricheva et al. 2004), as well as differences in herbivore pressure (Wetzel et al. 2023). Moreover, the conserved metabolic pathways involved in synthesizing specialized metabolites like phenolics and terpenoids (Zulak and Bohlmann 2010), likely contribute to similar defence induction patterns and resource allocation strategies (Moreira et al. 2013). Notably, in *P. pinaster*, these allocation constraints appeared consistently across different tissues and defensive traits—resin in stems and phenolics in needles—each linked to distinct herbivores and metabolic pathways (Franceschi et al. 2005; Mumm and Hilker 2006). Nevertheless, given the variability in costs and plasticity of various defensive traits (Bonello et al. 2006; Cipollini et al. 2014), further studies examining constitutive–induced defence correlations across additional traits are needed to fully characterize the defensive phenotype in this and other tree species.

Our study adds a novel dimension by showing that correlations between constitutive and induced defensive strategies depend strongly on environmental context. Specifically, we provide evidence that soil phosphorus availability—a key limiting factor in *Pinus pinaster* coastal ecosystems (Martíns et al. 2009)—modulates these defensive trade-offs. Under low phosphorus conditions, seedlings exhibited a strong negative correlation between constitutive and induced defences for both stem resin concentration and needle phenolics, whereas no significant association was found under high phosphorus availability. These results offer mechanistic support for resource allocation constraints as the primary driver of these negative correlations across both trait and organ types (for growth-defence trade-offs, see Fine et al. 2006; Moreira et al. 2014; Hahn and Maron 2016; López-Goldar et al. 2020), although alternative explanations such as pleiotropy cannot be fully excluded (Karasov et al. 2017). These context-dependent patterns have important ecological and evolutionary implications for *P. pinaster* in Galicia’s coastal range, where soil fertility and herbivory regimes vary significantly (Zas 2003; Zas et al. 2006, 2011; Martíns et al. 2009). In nutrient-poor environments, trees may face constraints that limit their ability to maintain both constitutive and induced defences, potentially increasing vulnerability to pest outbreaks if herbivore pressure intensifies. Consequently, forest management strategies should incorporate soil nutrient status when planning reforestation and pest control programs. Targeted phosphorus amendments might alleviate resource allocation constraints and bolster defensive capacity but must be applied cautiously to prevent nutrient leaching and unintended shifts in competitive dynamics. Integrating nutrient management with pest monitoring will likely be critical for sustaining forest health, particularly under the growing impacts of climate change on nutrient cycling and herbivore pressures in coastal forest ecosystems.

Further basic research—which will, in turn, help inform applied practices—should explore defensive strategies in *P. pinaster* through combined manipulations of phosphorus, nitrogen, and other interacting factors such as drought or salinity. Within this context, detailed studies aimed at quantifying the costs of different defensive traits and types of herbivore damage can provide predictive insights into abiotically driven defensive allocation constraints. Relatedly, a better understanding of endogenous (genetic and metabolic) linkages between defensive traits is needed, for example, using transcriptomic and metabolomic tools. Lastly, comparative studies across taxa with contrasting functional strategies, combined with nutrient manipulations, can enhance our understanding of how plant life-history traits and resource limitations shape defensive trade-offs and their ecological and evolutionary significance.

## Supplementary Information

Below is the link to the electronic supplementary material.

Supplementary file1 (DOCX 3383 KB)

## Data Availability

Data for peer review can be found online at figshare (Moreira et al. 2024).

## Abbreviations

JA: Jasmonic acid
P: Phosphorus availability

## References

[CR1] Agrawal AA, Hastings AP (2019) Trade-offs constrain the evolution of an inducible defense within but not between plant species. Ecology 100:e02857 31365759 10.1002/ecy.2857

[CR2] Bingham RA, Agrawal AA (2010) Specificity and trade-offs in the induced plant defence of common milkweed *Asclepias syriaca* to two lepidopteran herbivores. J Ecol 98(5):1014–1022

[CR3] Bonello P, Gordon TR, Herms DA, Wood DL, Erbilgin N (2006) Nature and ecological implications of pathogen-induced systemic resistance in conifers: a novel hypothesis. Physiol Mol Plant Pathol 68:95–104

[CR4] Bucci G, González-Martínez SC, Le Provost G, Plomion C, Ribeiro MM, Sebastiani F, Alía R, Vendramin GG (2007) Range-wide phylogeography and gene zones in *Pinus pinaster* Ait. revealed by chloroplast microsatellite markers. Mol Ecol 16(10):2137–2153 17498237 10.1111/j.1365-294X.2007.03275.x

[CR5] Cipollini D, Walters D, Voelckel C (2014) Costs of resistance in plants: from theory to evidence. Annual Plant Rev 47:263–307. 10.1002/9781119312994.apr0512

[CR6] Cole CT, Morrow CJ, Barker HL, Rubert-Nason KF, Riehl JFL, Köllner TG, Lackus ND, Lindroth RL (2021) Growing up aspen: ontogeny and trade-offs shape growth, defence and reproduction in a foundation species. Ann Bot 127:505–517 32296821 10.1093/aob/mcaa070 PMC7988516

[CR7] Coley PD, Bryant JP, Chapin FS (1985) Resource availability and plant antiherbivore defense. Science 230:895–899 17739203 10.1126/science.230.4728.895

[CR8] Endara MJ, Coley PD (2011) The resource availability hypothesis revisited: a meta-analysis. Funct Ecol 25:389–398

[CR9] Fine PV, Miller ZJ, Mesones I, Irazuzta S, Appel HM, Stevens MH, Sääksjärvi I, Schultz JC, Coley PD (2006) The growth-defense trade-off and habitat specialization by plants in Amazonian forests. Ecology 87(7):150–162 10.1890/0012-9658(2006)87[150:tgtahs]2.0.co;2 16922310

[CR10] Franceschi V, Krokene P, Christiansen E, Krekling T (2005) Anatomical and chemical defenses of conifer bark against bark beetles and other pests. New Phytol 167:353–376 15998390 10.1111/j.1469-8137.2005.01436.x

[CR11] Futuyma DJ, Agrawal AA (2009) Macroevolution and the biological diversity of plants and herbivores. Proc Natl Acad Sci U S A 106:18054–18061 19815508 10.1073/pnas.0904106106 PMC2775342

[CR12] Hahn PG, Maron JL (2016) A framework for predicting intraspecific variation in plant defense. Trends Ecol Evol 31:646–656 27282932 10.1016/j.tree.2016.05.007

[CR13] Kalske A, Kessler A (2023) Herbivory selects for tolerance and constitutive defence across stages of community succession. Proc R Soc Lond B Biol Sci 290:20222458 10.1098/rspb.2022.2458 PMC9928524 36787795

[CR14] Karasov TL, Chae E, Herman JJ, Bergelson J (2017) Mechanisms to mitigate the trade-off between growth and defense. Plant Cell 29:666–680 28320784 10.1105/tpc.16.00931 PMC5435432

[CR15] Kempel A, Schadler M, Chrobock T, Fischer M, van Kleunen M (2011) Tradeoffs associated with constitutive and induced plant resistance against herbivory. Proc Natl Acad Sci U S A 108:5685–5689 21389269 10.1073/pnas.1016508108 PMC3078369

[CR16] Kergoat GJ, Meseguer AS, Jousselin E (2017) Evolution of plant–insect interactions: insights from macroevolutionary approaches in plants and herbivorous insects. Adv Bot Res 81:25–53

[CR17] Koricheva J, Nykanen H, Gianoli E (2004) Meta-analysis of trade-offs among plant antiherbivore defenses: are plants jacks-of-all-trades, masters of all? Am Nat 163:64–75 10.1086/382601 15122510

[CR18] Labandeira C (2007) The origin of herbivory on land: initial patterns of plant tissue consumption by arthropods. Insect Sci 14:259–275

[CR19] Littell RC, Milliken GA, Stroup WW, Wolfinger R, Schabenberger O (2006) SAS System for mixed models, 2nd edition. Cary, NC, USA

[CR20] López-Goldar X, Zas R, Sampedro L (2020) Resource availability drives microevolutionary patterns of plant defences. Funct Ecol 34:1640–1652

[CR21] Martíns P, Sampedro L, Moreira X, Zas R (2009) Nutritional status and genetic control of phenotypic plasticity to nutrient availability in *Pinus pinaster*. A multisite field study in NW Spain. For Ecol Manage 258(7):1429–1436

[CR22] Massad TJ (2013) Ontogenetic differences of herbivory on woody and herbaceous plants: a meta-analysis demonstrating unique effects of herbivory on the young and the old, the slow and the fast. Oecologia 172:1–10 23053231 10.1007/s00442-012-2470-1

[CR23] de la Mata R, Lario FJ, Zas R (2024) Genetic and environmental considerations for the utilization of *Pinus pinaster* Ait. provenances across a region lacking proper local genetic materials. For Ecol Manage 570:122219

[CR24] Moreira X, Sampedro L, Zas R (2009) Defensive responses of *Pinus pinaster* seedlings to exogenous application of methyl-jasmonate: concentration effect and systemic response. Environ Exp Bot 67:94–100

[CR25] Moreira X, Lundborg L, Zas R, Carrillo-Gavilán A, Borg-Karlson AK, Sampedro L (2013) Inducibility of chemical defences by two chewing insect herbivores in pine trees is specific to targeted plant tissue, particular herbivore and defensive trait. Phytochemistry 94:113–122 23768645 10.1016/j.phytochem.2013.05.008

[CR26] Moreira X, Mooney KA, Rasmann S, Petry WK, Carrillo-Gavilán A, Zas R, Sampedro L (2014) Trade-offs between constitutive and induced defences drive geographical and climatic clines in pine chemical defences. Ecol Lett 17:537–546 24818235 10.1111/ele.12253

[CR27] Moreira X, Sampedro L, Zas R (2024) Dataset. Figshare. 10.6084/m9figshare/27633051v1

[CR28] Morris WF, Traw MB, Bergelson J (2006) On testing for a tradeoff between constitutive and induced resistance. Oikos 112(1):102–110

[CR29] Mumm R, Hilker M (2006) Direct and indirect chemical defence of pine against folivorous insects. Trends Plant Sci 11(7):351–358 16769239 10.1016/j.tplants.2006.05.007

[CR30] Núñez-Farfán J, Fornoni J, Valverde PL (2007) The evolution of resistance and tolerance to herbivores. Annu Rev Ecol Evol Syst 38:541–566

[CR31] Rasmann S, Agrawal AA (2011) Latitudinal patterns in plant defense: evolution of cardenolides, their toxicity, and induction following herbivory. Ecol Lett 14:476–483 21371232 10.1111/j.1461-0248.2011.01609.x

[CR32] Rasmann S, Chassin E, Bilat J, Glauser G, Reymond P (2015) Trade-off between constitutive and inducible resistance against herbivores is only partially explained by gene expression and glucosinolate production. J Exp Bot 66:2527–2534 25716695 10.1093/jxb/erv033 PMC4986863

[CR33] Runyon JB, Bentz BJ, Qubain CA (2022) Constitutive and induced defenses in long-lived pines do not trade off but are influenced by climate. J Chem Ecol 48:746–760 35982356 10.1007/s10886-022-01377-z

[CR34] Sampedro L, Moreira X, Zas R (2011) Costs of constitutive and herbivore-induced chemical defenses in pine trees emerge only under low resources availability. J Ecol 99:818–827

[CR35] Stamp N (2003) Out of the quagmire of plant defense hypotheses. Q Rev Biol 78:23–55 12661508 10.1086/367580

[CR36] Villari C, Faccoli M, Battisti A, Bonello P, Marini L (2014) Testing phenotypic trade-offs in the chemical defence strategy of Scots pine under growth-limiting field conditions. Tree Physiol 34:919–930 25194142 10.1093/treephys/tpu063

[CR37] Wetzel WC, Inouye BD, Hahn PG, Whitehead SR, Underwood N (2023) Variability in plant–herbivore interactions. Annu Rev Ecol Evol Syst 54:451–474

[CR38] Zas R (2003) Foliar nutrient status and tree growth response of young *Pseudotsuga menziesii* Mirb. (Franco) to nitrogen, phosphorus and potassium fertilization in Galicia (Northwest Spain). For Syst 12(1):75–85

[CR39] Zas R, Sampedro L, Prada E, Lombardero MJ, Fernández-López J (2006) Fertilization increases *Hylobius abietis* L. damage in *Pinus pinaster* Ait. seedlings. For Ecol Manage 222(1–3):137–144

[CR40] Zas R, Moreira X, Sampedro L (2011) Tolerance and induced resistance in a native and an exotic pine species: relevant traits for invasion ecology. J Ecol 99(6):1316–1326

[CR41] Zhang P-J, Shu J-P, Fu C-X, Zhou Y, Hu Y, Zalucki MP, Liu S-S (2008) Trade-offs between constitutive and induced resistance in wild crucifers shown by a natural, but not an artificial, elicitor. Oecologia 157(1):83–92 18491145 10.1007/s00442-008-1060-8

[CR42] Zulak KG, Bohlmann J (2010) Terpenoid biosynthesis and specialized vascular cells of conifer defense. J Integr Plant Biol 52(1):86–97 20074143 10.1111/j.1744-7909.2010.00910.x

[CR43] Züst T, Agrawal AA (2017) Trade-offs between plant growth and defense against insect herbivory: an emerging mechanistic synthesis. Annu Rev Plant Biol 68:513–534 28142282 10.1146/annurev-arplant-042916-040856

