# Supplementary material for: Soil phosphorus mediates trade-offs between constitutive and induced defences in young pine trees
Source: Planta. 2025 Aug 28;262(4):91. doi: 10.1007/s00425-025-04813-y (PMC12394365; doi:10.1007/s00425-025-04813-y)
Supplement: Supplementary file 1 — Supplementary file1 (DOCX 3383 KB) [file 425_2025_4813_MOESM1_ESM.docx]

**Figure S1.** Picture illustrating height differences between Pinus *pinaster* seedlings grown under high versus low phosphorus availability in the greenhouse.

**Explanations and annotated SAS code of the Monte Carlo simulation procedure used for distinguish between true trade-offs and spurious correlations**

We used the approach originally proposed by Morris et al. [1] to explore the true significance of the relationship between two variables, Y and X, when X is used to calculate Y as Y = Z – X. Since X is part of both the dependent and independent variables, Y and X tend to be spuriously (negatively) correlated. To distinguish between true trade-offs and spurious correlations, Morris et al. (2006) developed a Monte Carlo simulation procedure that accounts for this mathematical artefact. Here, we present the translation of the original MATLAB code into SAS language.

**(A) Simplified explanations of the simulation procedure**

**Fig. S2** Flowchart of the Monte Carlo approach used to distinguish between spurious correlations and the real trade-off between constitutive and induced defences in maritime pine families. For each family, induced defence was estimated as the difference between the mean phenotypic expression of the defensive trait after jasmonic acid (JA) induction and the mean value in control (constitutive) plants of the same family.


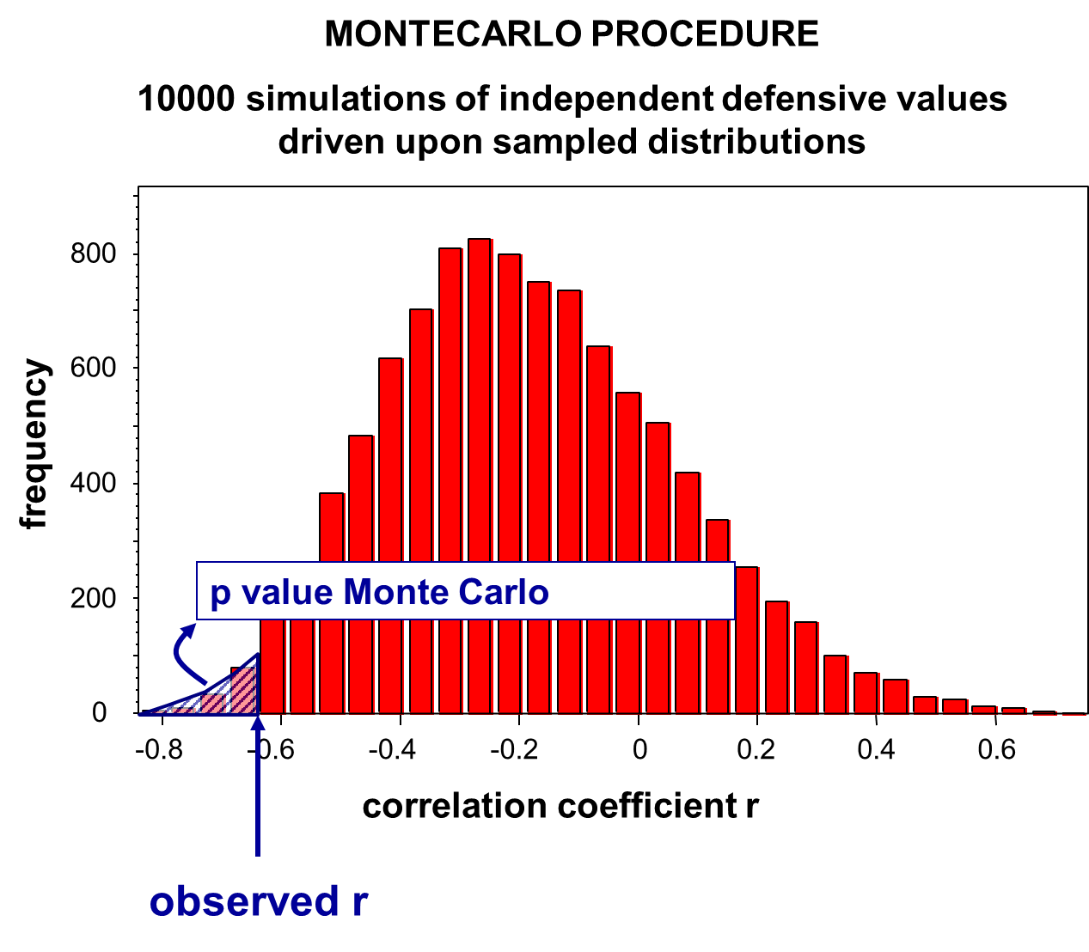


**Fig. S3** The main outcome of the Monte Carlo approach was used to distinguish between spurious correlations and the real trade-off between constitutive and induced defences in maritime pine families. The correlation coefficients from 10,000 simulations of independent constitutive and induced defence values, derived from the observed distributions, were calculated and plotted as a frequency histogram. The observed correlation coefficient is marked along this histogram, and the proportion of simulations with correlation coefficients lower than the observed coefficient was used as an empirical estimate of the probability of incorrectly assuming a significant correlation—i.e., the Monte Carlo p-value for a true trade-off.

**B) Annotated SAS code**

**data** TOF; * The dataset of least square means for each family in each fertilization treatment and each induction treatment;

length T $ **5** G $ **8** JA $ **3**;

infile 'C:\...\TOFPue.csv' delimiter=';' firstobs=**2** dsd;

input T JA G NVR PHE SENVR SEPHE;

resistanttrait=PHE;

if T='P-DEF';

**run**;

* T is the fertilization treatment: P-DEF or P-COMplete;

* G is the family (33 levels);

* JA is the induction treatment: CTR and JA-induced;

* PHE and NVR are the defensive trait: the phenolics and non-volatile resin means for each family;

* SEPHE and SENVR are the standard errors of family means;

%let nfam=33; * NFAM = number of families or genetic entries;

%let n=4; * n = number of replicates per family;

%let repeticiones=10000; * Number of Montecarlo simulations */

**data** MeanF2; /*induced=damage-control*/

merge TOF (where=(JA=**0**) rename=(resistanttrait=C) Keep=G resistanttrait JA)

TOF (where=(JA=**1**) rename=(resistanttrait=D) Keep=G resistanttrait JA);

by G;

I=D/C;

**run**;

**proc** **means** noprint; /*Grand means and variance of family means*/

var C I;

output out=GrandMean mean=MC MI var=VC VI LCLM=lMC lMI UCLM=uMC uMI stderr=SEC SEI;

**proc** **means** data=TOF noprint; /*Computes mean within family variances*/

by JA;

var SE;

output out=MeanV mean=MeanSE;

**data** _null_;

set MeanV;

if JA=**0** then call symput('MeanVC',MEANSE****2**);

if JA=**1** then call symput('MeanVD',MEANSE****2**);

**data** RESULTS;

**%macro** ***Montecarlo***;

options nonotes mautosource nomprint nosource nodate;

%do step=**1** %to &repeticiones;

%put Estamos en el paso &step;

data grandmeansim; /*randomized simulated values within the 95% confidence Interval of means and variances for both constitutive and induced levels;

set grandmean;

MCsim=**0**;VCsim=**0**;MIsim=**0**;VIsim=**0**;

LVC=VC*(&nfam-**1**)/CINV(**0.975**,&nfam-**1**); UVC=VC*(&nfam-**1**)/CINV(**0.025**,&nfam-**1**);

LVI=VI*(&nfam-**1**)/CINV(**0.975**,&nfam-**1**); UVI=VI*(&nfam-**1**)/CINV(**0.025**,&nfam-**1**);

do until(MCsim<uMC and MCsim>lMC);

MCsim = MC+SEC*rand('NORM');

call symput('MCsim',MCsim);

end;

do until(VCsim<uVC and VCsim>lVC);

VCsim = VC*RAND('CHIS',&nfam-**1**)/&Nfam;

call symput('VCsim',VCsim);

end;

do until(MIsim<uMI and MIsim>lMI);

MIsim = MI+SEI*rand('NORM');

call symput('MIsim',MIsim);

end;

do until(VIsim<uVI and VIsim>lVI);

VIsim = VI*RAND('CHIS',&nfam-**1**)/&Nfam;

call symput('VIsim',VIsim);

end;

keep MCsim MIsim VCsim VIsim;

data MeanFsimC; set grandmeansim;

call streaminit(&step+**10**);

m=log(MCsim****2**/sqrt(VCsim+MCsim****2**));

s=sqrt(log(VCsim/MCsim****2**+**1**));

Cmin=MCsim;

do NF=**1** to &nfam;

C=exp(m+s*rand('NORM'));output;

if Cmin>C then Cmin=C;

end;

call symput('Cmin',Cmin);

keep NF C;

data MeanFsimI; set grandmeansim;

call streaminit(&step+**100**);

m=log((MIsim+&Cmin)****2**/sqrt(VIsim+(MIsim+&Cmin)****2**));

s=sqrt(log(VIsim/(MIsim+&Cmin)****2**+**1**));

do NF=**1** to &nfam;

I=exp(m+s*rand('NORM'))-&Cmin;output;

end;

keep NF I;

data Datasim;

merge MeanFsimC MeanFsimI;

by NF;

call streaminit(&step);

D=I*C;

mC=log(C****2**/sqrt(&MeanVC+C****2**));

sC=sqrt(log(&MeanVC/C****2**+**1**));

mD=log(D****2**/sqrt(&MeanVD+D****2**));

sD=sqrt(log(&MeanVD/D****2**+**1**));

do j=**1** to &n;

Cj=exp(mC+sC*rand('NORM'));

Dj=exp(mD+sD*rand('NORM'));

output;

end;

proc means noprint;

by NF;

var Cj Dj;

output out=MeanSim mean=Csim Dsim;

data MeanSim;

set MeanSim;

Isim=Dsim/Csim;

proc corr data=MeanSim outP=r noprint;

var Csim Isim;

data r; set r;if _name_='Csim';keep _name_ Isim;

data RESULTS; set RESULTS r;run;

%end;

**%mend**;

%***Montecarlo***

**proc** **gchart** data=RESULTS; vbar Isim;**run**;**quit**;

**proc** **means** data=RESULTS mean std P10 P5 P1 min;var Isim;**run**;

**proc** **corr** data=meanf2; var C I;**run**;

**proc** **sort** data=results; by Isim;**run**;

**References**

Morris WF, Traw MB, Bergelson J (2006) On testing for a tradeoff between constitutive and induced resistance. Oikos 112: 102-110
